# Coherence Time Evaluation in Indoor Optical Wireless Communication Channels

**DOI:** 10.3390/s20185067

**Published:** 2020-09-07

**Authors:** Dima Bykhovsky

**Affiliations:** Shamoon College of Engineering, Beer-Sheva 8410802, Israel; dmitrby@sce.ac.il

**Keywords:** optical wireless communication, coherence time, optical wireless channel, autocovariance function (ACF), nine degrees of freedom (9DoF)

## Abstract

The coherence time is the time over which the channel-gain-values correlation coefficient drops below a predefined threshold. The coherence time is typically used to quantify the pace of appreciable channel changes and is important, for example, for determining handoff and resource allocation time constraints. The goal of this work is to experimentally measure the coherence time of indoor optical wireless communication (OWC) channels under various mobile scenarios. The amount of movement was quantified by mobile sensor measurements. The experiments show that it is reasonable to assume that the channel varies slowly for a time period of ~100 milliseconds for most mobile scenarios.

## 1. Introduction

Commonly used descriptions of the mobile fading channel include coherence metrics, namely, coherence bandwidth, coherence distance, and coherence time [1]. In optical wireless communication (OWC), the coherence bandwidth is related to the multiple reflections that may be associated with a communication signal and quantitatively characterizes the amount of the multipath effect and its possible influence on wide-band communication [2,3,4,5]. The coherence distance is the distance between transceiver locations over which the channel gain does not change appreciably [6]. This metric is mainly related to the surrounding indoor environment. The coherence time is the characteristic time over which the channel gain does not change appreciably. This metric is mainly influenced by mobile transceiver mobility and is significantly related to users’ movement [1].

The analysis of indoor mobile channels is challenging for several reasons. First, it involves numerous factors such as mobile device orientation [7,8,9,10,11,12], distance dependency [13], shadowing [14,15,16], and others. Another aspect is related to reliable simulation of human movement; while realistic human motion simulations are possible, their combination with rigorous channel characterization is still challenging [17,18]. The current state-of-the-art results are based on the experimental study of mobile users’ behavior and the development of a statistical model for the orientation of mobile devices; this model further serves as a base for extensive simulation analysis [10,11,12]. The results also include closed-form expressions for indoor OWC channel statistics that address both the user’s mobility and the random device orientation [12]. Nevertheless, the previously reported experimental studies of mobile user behavior overlook the aspect of time-domain analysis of channel changes.

This research concentrates on the experimental analysis of mobile user behavior, with emphasis on time-related changes. The experiments served to characterize the pace of channel changes, with coherence time used as a metric. In particular, this metric may be useful to indicate the amount of pilot symbols required to provide sufficient channel estimation accuracy and to define a recalculation rate requirement for multiuser resource allocation schemes [19]. Different user mobility scenarios were evaluated. In order to quantify the mobility, the measurements from a 3-axis accelerometer, 3-axis gyroscope, and 3-axis magnetometer (also termed a nine degrees of freedom (9DoF) sensor) were applied and their relation with coherence time was established.

The rest of the paper is organized as follows. The pertinent theory regarding OWC channels and the coherence time are provided in Section 2. Section 3 presents the experimental set-up and Section 4 analyzes the resulting experimental results. Discussion of the results is provided in Section 5, and Section 6 summarizes and concludes.

## 2. Theory

The channel state of interest represents the steady-state DC gain between the transmitter and the receiver. It includes both line-of-sight (LOS) and non-LOS (NLOS) components, while the possible intersymbol interference (ISI) effect is neglected and assumed to be fully mitigated by proper equalization, e.g., by use of an appropriate optical orthogonal frequency-division modulation (OFDM) scheme [20].

### 2.1. Transceiver Distance and Device Orientation

Communication through optical channels mainly relies on line-of-sight (LOS) links that are significantly dependent on the separation distance and axial misalignment of a transmitter–receiver link. The simplified channel gain attenuation model goes as $∼d^{-\gamma }$, where *d* is transceiver separation and the factor γ varies between 2 for axially aligned links up to about 4 for a significant misalignment or non-LOS conditions [21]. While some previous studies have assumed that the receiver faces vertically upward and is randomly located within the coverage area, it was recently shown that this is not a realistic assumption from a practical point of view [10,11,12]. Additional factors, such as room configuration, transmitter lighting profile, wall reflections, and shadowing, are considered to be less influential [14,15,16,17,18].

### 2.2. Coherence Time

The channel gain is modeled by a stationary discrete-time continuous-valued random process, x[n]. The derivation is based on an auto-covariance function (ACF) of the form

(1) $$C_{x}\left[k\right]=E\left[x[n]x[n+k]\right]-E\left[x^{2}\left[n\right]\right],$$

where E[·] stands for the arithmetic mean. The corresponding normalized temporal correlation coefficient is given by

(2) $$\rho_{x}\left[k\right]=\frac{C_{x}\left[k\right]}{C_{x}\left[0\right]},$$

and describes the accuracy of a minimum mean square error (MMSE) prediction of x[n] [22]. The resulting coherence time, $k_{c}$, is given by the solution of

(3) $$\rho_{h}\left[k_{c}\right]=\rho_{thr},$$

where common $\rho_{thr}$ values are 0.1, exp(−1), 0.5, and 0.9 [6,23]. Throughout the paper, $\rho_{thr}=0.5$ was applied.

The main accuracy limitation of the described procedure is the requirement that $k_{c}\ll N$, where *N* is the number of channel samples. The reason for this requirement is the fact that $\rho_{h}\left[k\right]\to 0$ when k→N and $\rho_{h}\left[k\right]=0$ for k>N. The evaluated value of coherence time may be easily converted to continuous-time units (i.e., seconds) by the relation $\tau_{c}=k_{c}/f_{s}$, where $f_{s}$ is the channel sampling frequency (measured in Hz).

## 3. Experimental Setup

### 3.1. Light Emitting Diode (LED)

**Figure 1:**
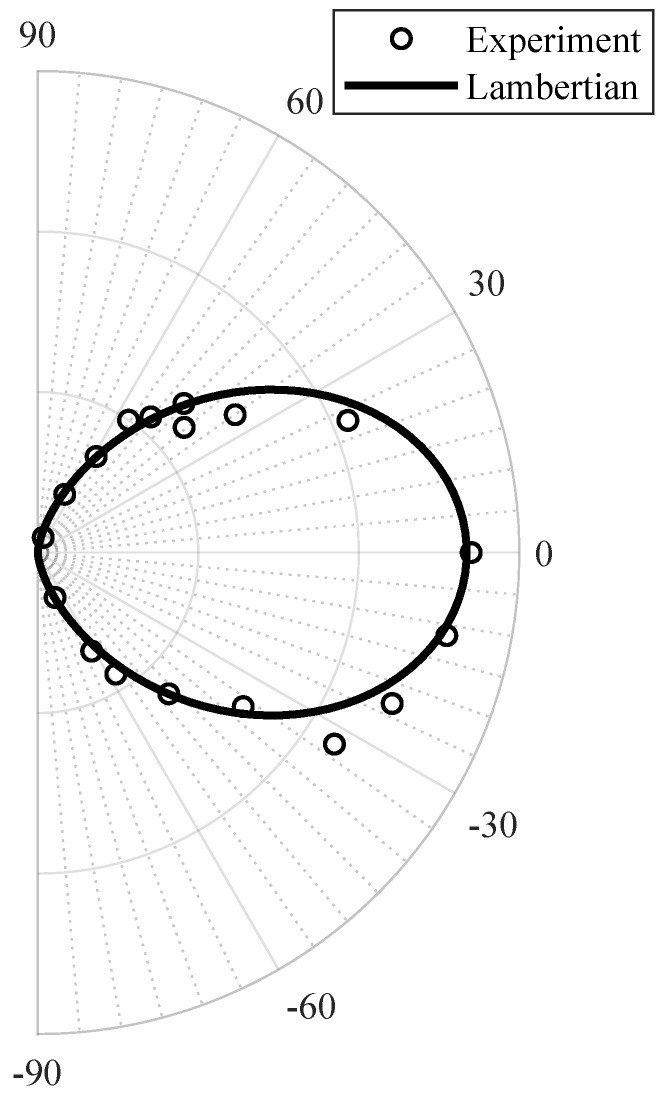
The fit of the Lambertian model with m=2.1.

The transmitter is modeled by a light-emitting diode (LED) lamp. In order to prevent flickering resulting from the electrical network frequency and its harmonics, a battery-powered LED was applied. The LED was modeled by a modified Lambertian profile (measured in W/sr) of the form [24]

(4) $$I\left(\phi \right)∼\frac{2\pi }{m+1}\cos^{m}\left(\phi \right),$$

where ϕ is the off-normal angle of emission relative to the optical axis and *m* is the order of the model. The fit of the experimental measurements to the applied LED model is presented in Figure 1 with the value of m=2.1 that illustrates intermediate directionality.

### 3.2. Measurements

The received signal level was acquired by a photodetector and the output current was digitized at a 100 Hz sampling frequency and recorded for further off-line analysis with Thorlabs PM400K3 power meter with S130C power sensor. The resulting coherence time was evaluated using Equation (3).

**Figure 2:**
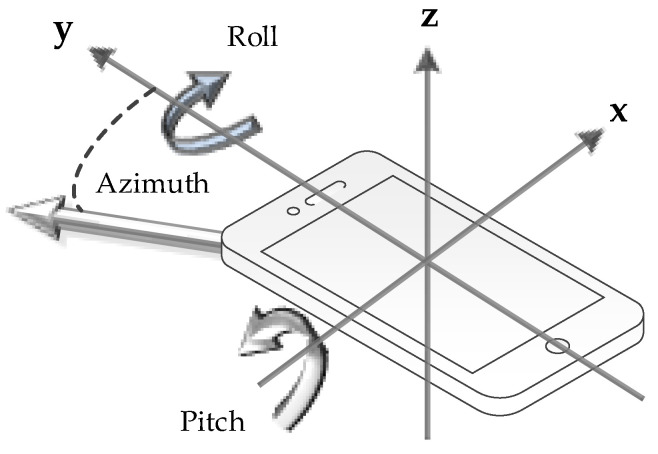
Visualization of the local axes and rotation angles, i.e. roll, pitch and azimuth.

The mobile sensor measurements were provided by a smartphone (Mi Note 8) that was physically coupled with the photodetector and were sampled at 100 Hz and logged by the “Matlab Mobile” application for Android. The following sensor measurements were logged: acceleration (measured in m/s^2^); orientation, i.e., azimuth, pitch, and roll angles (measured in ${}^{\circ }$); and angular velocity (measured in rad/s). All logged sensor measurements are relative to the local coordinate system as outlined in Figure 2.

### 3.3. Scenarios

The experimental scenarios address the type of user behavior that reflects fast and significant changes in channel gain and include walking, standing up, sitting down, and rotating. These are in contrast to the slow changes in receiver location and/or environment that result in relatively slow changes in the channel gain.

**Figure 3:**
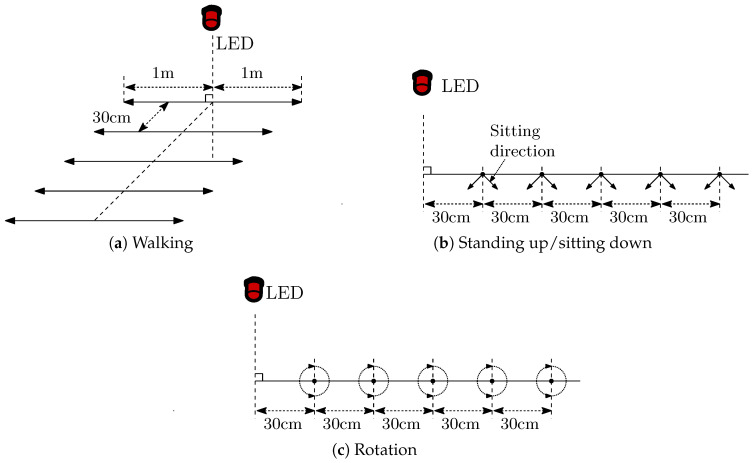
Scenarios for channel measurements.

- Walking: The main expected reason for channel changes is a change in transceiver separation distance. The experimental scenario realization is presented in Figure 3a. In this scenario, the user walks a distance of approximately 2 m, in a horizontal plane below the signal source. The horizontal displacement is shifted by 30 cm away from the signal source between subsequent measurement sets, with ten measurements in each set, making a total of 50 measurements.
- Standing up/sitting down: During these operations a receiver experiences rapid distance, orientation, and shadowing changes at once. In this scenario, standing up and sitting down are performed at two different orientations and five different horizontal positions spaced 30 cm from one another, with each measurement performed twice (Figure 3b), making a total of 20 measurements for each operation.
- Rotating: In this case, the main reasons for channel changes are orientation and shadowing. In this scenario the measurements included rotations of 360${}^{\circ }$, twice, with five different horizontal positions spaced 30 cm from one another (Figure 3c). The measurements were repeated for both standing, with approximately 120 cm vertical distance between the lamp and the receiver, and sitting on a rotating chair, with approximately 165 cm vertical distance between the lamp and the receiver, making a total of 40 measurements.

A summary of the physical parameters of the considered experimental set-up is presented in Table 1.

## 4. Experimental Results

The goal of this section is to describe and analyze the time-domain experimental measurements of indoor OWC gain under various behavioral conditions.

### 4.1. Illustrative Result

**Figure 4:**
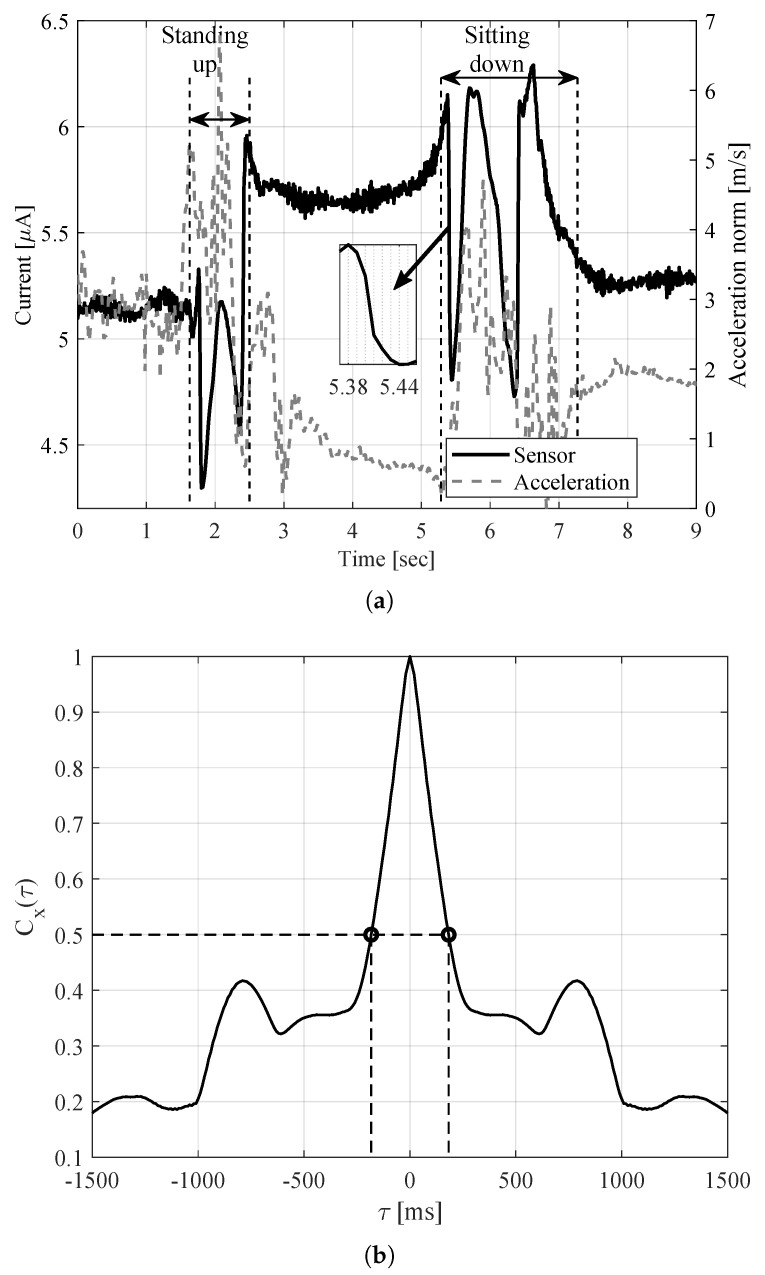
Illustrative example of channel behavior. (**a**) The received signal level with the corresponding acceleration vector norm. (**b**) The normalized auto-covariance function (ACF) of the received signal with coherence time of 183 ms.

In order to illustrate the relation between sensor measurements, the following example presents the standing up motion followed by sitting down. Figure 4a shows the received signal level with the corresponding linear acceleration vector norm as recorded by mobile sensors. A sample measurement of the fast change of the received signal is expanded in the inset. The corresponding normalized ACF in Figure 4b reflects the coherence time of ~183 ms.

### 4.2. Characterization

The characterization parameters that were further applied for evaluating the coherence time are summarized in Table 2. These parameters were either collected by sensors or directly derived from sensor measurements by transformation between local and global (Earth-related) coordinate systems, as detailed in Appendix A.

The following characterization metrics were applied for each parameter from Table 2,

(a) maximum change, i.e., difference between highest and lowest value;
(b) standard deviation;
(c) peak Fourier transform amplitude of non-DC component; and
(d) frequency of the peak Fourier transform amplitude of non-DC component.

The matrix of cross-covariances between the characterization metrics and coherence times was evaluated and the results are summarized in Table 3. Only statistically significant matrix values with a *p*-value lower than 0.01 are presented.

**Figure 5:**
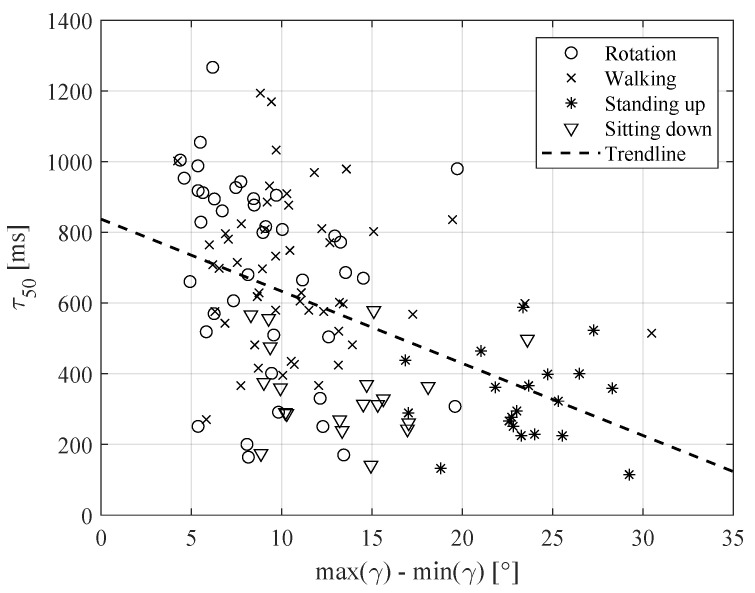
Relation between coherence time and maximum change in local roll angle (metric (a), parameter #2).

**Figure 6:**
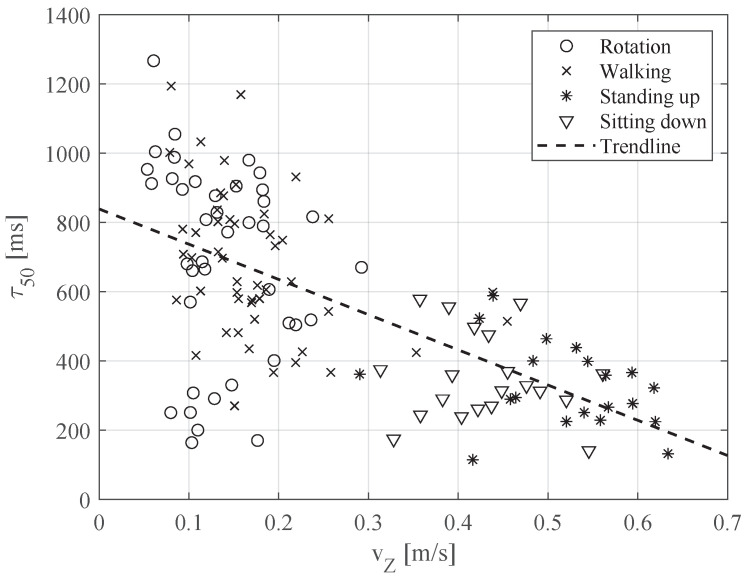
Relation between coherence time and peak amplitude of Fourier transform of linear acceleration in local Z direction (metric (c), parameter #8).

For example, the relation between maximum change in local roll angle (metric (a), parameter #2) and coherence time is presented in Figure 5, with a cross-correlation coefficient of −0.48 (see Table 3), while the higher change in roll angle resulted in faster channel changes and, as a result, in a lower coherence time value. Another example, of the relation between the peak amplitude of the Fourier transform of the linear acceleration in the local Z direction (metric (c), parameter #8) and the coherence time, with a cross-correlation coefficient of −0.63 (see Table 3), is presented in Figure 6, where the higher peak amplitude resulted in a low coherence time value. Further discussion of the results is provided in the following section.

## 5. Discussion

### 5.1. Characterization

The outcomes of some of the evaluated combinations of characterization parameters and metrics were very instructive, as outlined in the examples above. However, some of them were less useful. In particular, characterization parameters #1, 3, 5, 6, 9, 10, 15–18 for all metrics, and metric (d), do not exhibit any strong relation to the corresponding coherence times.

The difference between metrics (c) and (d) may be explained as follows. Both metrics (c) and (d) are based on the amplitude and frequency of the most significant non-DC Fourier transform component and both are related to the most significant movement, as recorded by the sensors. However, metric (c) is related to the amplitude of the movement and metric (d) is related to the time-length of the movement. Therefore, the results show that the amplitude of the movement has a more significant influence on the coherence time than its time-length. This conclusion also explains the similarity between metrics (a) and (c).

**Figure 7:**
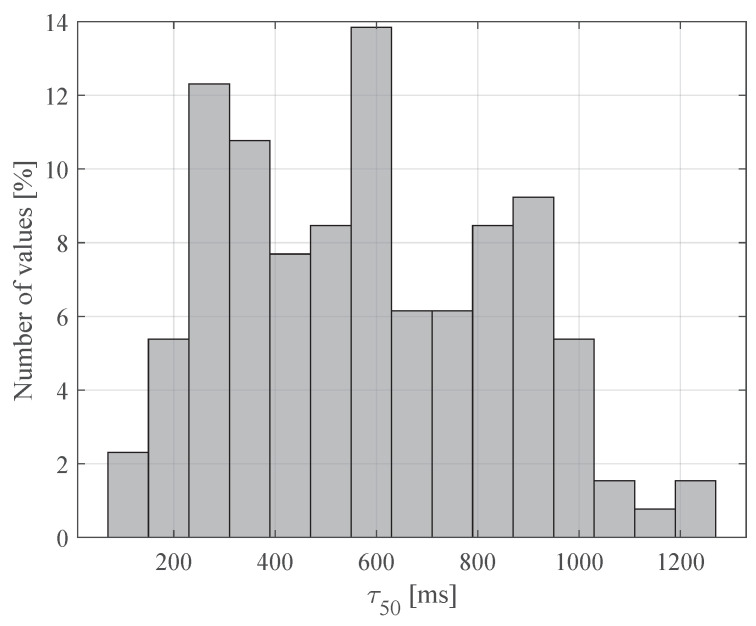
Histogram of the resulting coherence time values for all evaluated scenarios.

A histogram of the resulting coherence time values is presented in Figure 7. The coherence time values during the experiments are 150 ms or higher in 97.5% of measurements. Moreover, as most of the experimental results reflect rapid and significant motion, the expected coherence time may be in the order of seconds for slower motion.

### 5.2. Fast and Slow Fading

One of the important fading classifications is fast and slow fading conditions. This classification indicates how rapidly the transmitted signal changes as compared to the rate of changes of the channel [1]. In a slow fading channel, the channel changes significantly more slowly than the symbol rate of the signal, and is a common condition for high-speed transmissions. In a fast fading channel, the channel notably changes within the duration of a communication symbol, and is commonly related to the cases of low-power and energy-harvesting sensors [25,26,27]. As follows from the coherence time definition (Section 2.2), in such a channel the channel gain for the next symbol cannot be predicted from the previous one, as they are virtually uncorrelated [28]. This contrasts with slow fading conditions that can be easily mitigated by commonly deployed techniques, such as pilot-based channel prediction.

The symbol rates of about $T_{s}\gg 1/\tau_{c}$ and higher may be considered as slow-faded [1]. By following the experimental results and substituting the minimum experimental coherence time value, the lowest recommended symbol rate is about 100 symbol/s; this symbol rate range should sufficiently enable effective mitigation of the detrimental effects due to fading.

The influence of coherence time may also be studied from an information theory point of view. The capacity of a fading channel is directly related to the coherence time, which characterizes the length of the time interval usable for channel estimation at the receiver, and hence the achievable channel estimation error [29]. For a sufficiently long coherence time (i.e., slow fading conditions) the channel capacity is close to the capacity with exact knowledge of the channel state. On the other hand, if the channel uncertainty is too large (i.e., fast fading), then the channel capacity is significantly smaller.

### 5.3. Threshold Values

**Figure 8:**
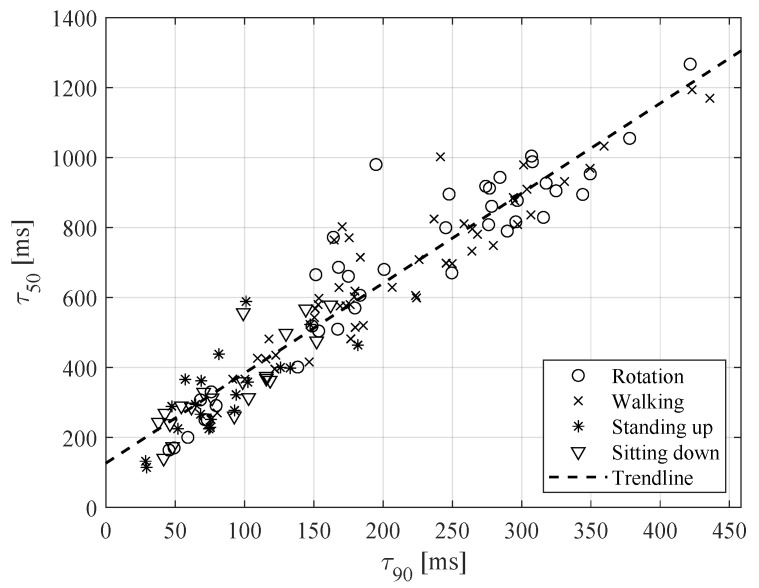
Relation between coherence times resulting from $\rho_{thr}=0.5$ and from $\rho_{thr}=0.9$ in Equation (3).

During the evaluation of coherence time values, the value of $\rho_{thr}=0.5$ was applied in Equation (3). Figure 8 shows the relation between coherence times resulting from $\rho_{thr}=0.5$ and from $\rho_{thr}=0.9$. The presented graph shows high resemblance between these times. This relation is important since the accuracy of ACF estimation decreases due to the time-limited nature of the signals, as outlined above in Section 2.2.

All the considered scenarios of walking, standing up, sitting down and rotating may produce rapid changes in the channel gain and the corresponding coherence time. Nevertheless, following the results displayed in Figure 8, walking seems to be related to the slowest gain changes.

## 6. Summary and Conclusions

The indoor OWC channel depends on the spatial location and orientation of a receiver, relative to a transmitter location and orientation, as well as on shadowing. Some of these factors may be efficiently characterized by sensors commonly present in smartphones, such as an accelerometer, gyroscope, and magnetometer. In this paper, the coherence time of an indoor OWC channel was sensor-characterized in different dense-motion scenarios. The experiments show that the resulting coherence time values were in the order of hundreds of milliseconds, with the lowest values being in the range of 100 to 150 milliseconds. Moreover, the coherence time may be effectively characterized by sensor measurements.

A natural progression of this work is to further analyze interrelations between the received signal levels and sensor measurements. This analysis may be useful for predicting a significant change in the channel gain, and may reveal the possibility of more accurate channel state prediction and pave the way towards improving link restoration after deep fading conditions.

## Figures and Tables

**Table 1:** Physical parameters of the experimental set-up.

Parameter	Value
Room height	2.4 m
Room width	4 m
Room length	3.5 m
Sampling frequency	100 Hz
Sensor measurements	acceleration, orientation, angular velocity
Walking distance	2 m
Chair height	53 cm

**Table 2:** Characterization parameters.

#	Measurement Type	Measurement	Note
1	Anglular position (orientation)	local azimuth	
2		local roll	
3		local pitch	
4		global polar	Equation (A3)
5		global azimuth	Equation (A4)
6	Linear Acceleration	local X	
7		local Y	
8		local Z	
9		global X	Equation (A1)
10		global Y	
11		global Z	
12		norm	with *g*
13		norm	without *g*
14	Angular Velocity	local azimuth	
15		local roll	
16		local pitch	
17		global polar	Equation (A5)
18		global azimuth	Equation (A6)

**Table 3:** Cross-covariance between characterization metrics and coherence times.

Parameter	Metric			
	(a)	(b)	(c)	(d)
#1	0.24	0.25	0.28	
#2	−0.48	−0.46	−0.39	
#3			−0.26	
#4	−0.47	−0.47	−0.40	
#5				
#6				0.39
#7	−0.49	−0.54	−0.49	
#8	−0.55	−0.59	−0.63	0.31
#9				
#10				
#11	−0.56	−0.57	−0.60	0.36
#12	−0.56	−0.57	−0.60	0.29
#13	−0.53	−0.58	−0.54	
#14	−0.43	−0.50	−0.49	0.28
#15				
#16	0.23			
#17				
#18	0.29	0.23		

## Appendix A. Local to Global Coordinate System Transformation

### Appendix A.1. Linear Acceleration

In order to rotate acceleration vector, n, with respect to the vertically upward coordinate system $n_{0}$, the Euler rotation may be applied by [30]

(A1) $$n_{0}=R_{\alpha }R_{\beta }R_{\gamma }n,$$

where

(A2a) $$\begin{array}{cc} R_{\alpha } & =\left[\begin{array}{ccc} 1 & 0 & 0\\ 0 & \cos(\alpha ) & -\sin(\alpha )\\ 0 & \sin(\alpha ) & \cos(\alpha ) \end{array}\right] \end{array}$$

(A2b) $$\begin{array}{cc} R_{\beta } & =\left[\begin{array}{ccc} \cos(\beta ) & 0 & \sin(\beta )\\ 0 & 1 & 0\\ -\sin(\beta ) & 0 & \cos(\beta ) \end{array}\right] \end{array}$$

(A2c) $$\begin{array}{cc} R_{\gamma } & =\left[\begin{array}{ccc} \cos(\gamma ) & -\sin(\gamma ) & 0\\ \sin(\gamma ) & \cos(\gamma ) & 0\\ 0 & 0 & 1 \end{array}\right] \end{array}$$

and α,β, and γ denote azimuth (yaw), pitch, and roll angles, respectively.

### Appendix A.2. Orientation

In order to convert local angles to global ones, the following conversion may be applied [30],

(A3) $$\begin{array}{cc} \theta & =\mathrm{acos}\left(\cos(\beta )\cos(\gamma )\right), \end{array}$$

(A4) $$\begin{array}{cc} \omega & =\mathrm{atan}(\frac{\sin(\alpha )\sin(\beta )\cos(\gamma )+\cos(\alpha )\sin(\gamma )}{\sin(\alpha )\sin(\gamma )-\cos(\alpha )\sin(\beta )\cos(\gamma )}), \end{array}$$

where θ is the polar angle and ω is the azimuth angle.

### Appendix A.3. Angular Acceleration

Global angular acceleration may be evaluated by differentiation of the orientation angles in the above, as

(A5) $$\begin{array}{cc} \dot{\theta } & =\frac{\dot{\beta }\sin\left(\beta \right)\cos\left(\gamma \right)+\dot{\gamma }\cos\left(\beta \right)\sin\left(\gamma \right)}{\sqrt{1-\cos^{2}\left(\beta \right)\cos^{2}\left(\gamma \right)}} \end{array}$$

(A6) $$\begin{array}{cc} \dot{\omega } & =-\dot{\alpha }+\frac{\dot{\beta }\cos\left(\beta \right)\sin\left(\gamma \right)\cos\left(\gamma \right)-\dot{\gamma }\sin\left(\beta \right)}{\sin^{2}\left(\beta \right)\cos^{2}\left(\gamma \right)+\sin^{2}\left(\gamma \right)}, \end{array}$$

where local angular acceleration is provided by sensor measurements.
